# Vitamin 25(OH)D_3_, E, and C Supplementation Impact the Inflammatory and Antioxidant Responses in Piglets Fed a Deoxynivalenol-Contaminated Diet and Challenged with Lipopolysaccharides

**DOI:** 10.3390/toxins16070297

**Published:** 2024-06-28

**Authors:** Béatrice Sauvé, Younes Chorfi, Marie-Pierre Létourneau-Montminy, Frédéric Guay

**Affiliations:** 1 Department of Animal Sciences, Laval University, Quebec, QC G1V 0A6, Canada; 2 Department of Veterinary Biomedicine, University of Montréal, Saint-Hyacinthe, QC J2S 2M2, Canada

**Keywords:** piglets, deoxynivalenol, oxidation, inflammation, vitamin E, vitamin C, vitamin 25(OH)D_3_, lipopolysaccharides

## Abstract

Using alternative ingredients or low-quality grain grades to reduce feeding costs for pig diets can introduce mycotoxins such as deoxynivalenol (DON) into feed, which is known to induce anorexia, inflammation, and oxidative stress. Adding vitamin 25(OH)D_3_ or vitamins E and C to the feed could increase piglets’ immune system to alleviate the effects of DON. This study used 54 pigs (7.8 ± 0.14 kg) in 27 pens (2 pigs/pen) with a vitamin 25(OH)D_3_ or vitamin E-C supplementation, or their combination, in DON-contaminated (5.1 mg/kg) feed ingredients over 21 days followed by a lipopolysaccharide (LPS) challenge (20 µg/kg BW) 3 h prior to euthanasia for 1 piglet per pen. DON contamination induced anorexia, which reduced piglet growth. DON also induced immunomodulation, oxidative stress, and downregulated vitamin D status. The vitamin E and C supplementation and the combination of vitamins E, C, and 25(OH)D_3_ provided protection against DON contamination by not only decreasing blood and liver oxidative stress markers, but also by increasing antioxidant enzymes and tocopherol levels in blood, indicating improved antioxidant defense mechanisms. The combination of vitamins also restored the vitamin D status. After LPS challenge, DON contamination decreased intestinal and liver antioxidant statuses and increased inflammation markers. The addition of vitamins E and C to DON-contaminated feed reduced markers of inflammation and improved the antioxidant status after the LPS immune stimulation. The combination of all these vitamins also reduced the oxidative stress markers and the inflammation in the intestine and mesenteric lymph nodes, suggesting an anti-inflammatory effect.

## 1. Introduction

Mycotoxins can be a challenge for swine producers, as it is often found in feed at different concentrations. One of the most common mycotoxins is deoxynivalenol (DON) synthesized by *Fusarium* fungi. This secondary metabolite belongs to the trichothecene group and is a chemical agent part of the sesquiterpenoid group [1]. The Canadian Food Inspection Agency and the US Food and Drug Administration have established a recommendation for maximum level at 1.0 mg/kg of DON in finished wheat products to avoid any health problems in animals [2,3]. In pigs, DON-contaminated feed ingredients at higher doses (3.0 mg/kg to 8.0 mg/kg of DON) caused partial anorexia [4,5], reduced growth, altered immune function [6], and induced oxidative stress [7]. DON has also been reported to result in upregulation of the gene expression related to pro-inflammatory cytokines, chemokines, and apoptosis, an effect that depends on the dose and exposure time [2]. It was also observed by Lessard et al. (2015) that antioxidant and inflammatory oxidative enzymes including nitric oxide synthase-2 (NOS_2_) were upregulated in jejunum of pigs with a DON contamination (3.5 mg/kg) in diets. Thus, this modulated immune and antioxidant responses, along with a reduced resistance toward pathogen-associated molecular patterns like lipopolysaccharides (LPS) [8]. As part of the outer membrane of Gram-negative bacteria, LPS are commonly found in the environment and intestinal microbiota of pigs [9,10]. Also, both DON and LPS have been shown to alter the intestinal barrier function, leading to an increased gut permeability and absorption of nutrients and toxins [11].

As DON modulates inflammatory response and oxidative stress, additives may be used in animal feed to control these metabolic pathways. Indeed, 25-hydroxyvitamin D_3_ (25(OH)D_3_), vitamin E, or vitamin C can be included in piglets feed to enhance their immune response and antioxidant status [12,13,14] during an immune challenge, such as DON exposure. Production of 25(OH)D_3_ comes from the hydroxylation of the vitamin D_3_ in the liver at the C-25 position by the 25-hydroxylase activity that is regulated by the CYP2R1 gene. Then, 25(OH)D_3_ reaches the kidney to be cleaved by the 1α-hydroxylase that is controlled by the CYP27B1 gene, at C-1 position, to make 1,25(OH)_2_D_3_, also called calcitriol. The latter is the active hormonal form of vitamin D, responsible for most of its biological actions [15,16]. As CYP27B1 and the vitamin D receptor (VDR) are both expressed in cells involved in immune and antioxidant functions, calcitriol likely plays a role in modulating the inflammatory and antioxidant responses. In fact, calcitriol can downregulate the immune system by inhibiting the production of pro-inflammatory cytokines in humans [14]. Vitamin D can also minimize the oxidative changes by reducing the production of reactive oxygen species (ROS), such as H_2_O_2_ and O_2_^−^, in diabetic rats and mice [17]. Finally, vitamin D, through VDR, has an important role in preserving the integrity of the gut membrane as a barrier and the tight junction complex [18]. Thus, the 25(OH)D_3_ supplementation could prevent many detrimental effects of DON.

Vitamin E, in its α-tocopherol form, is considered a liposoluble antioxidant synthesized by plants and an important constituent of cell membranes [13,19]. In the biological membranes, vitamin E protects their components from oxidation by free radicals, and thus oxidative damages [19]. As a chain-breaking antioxidant, vitamin E has a significant role in preserving the integrity of cell membranes [13], including that of the intestinal barrier, a target of DON [20]. In pigs (11.1 kg) receiving 4 mg/kg of DON-contaminated diet, the addition of vitamin E partially prevented oxidative stress-induced DNA fragmentation [21]. On the other hand, vitamin C, in its major form as ascorbic acid, is a water-soluble vitamin absorbed through passive pathways [22]. Vitamin C is considered an antioxidant, as it protects biomolecules from oxidative damage by ROS during exposure to toxins or pollutants. It can enhance the epithelial barrier function in part by increasing the expression of tight junction proteins. It also plays a role in the regulation of the immune response, as it influences the functions of neutrophils during inflammation [12]. Additionally, the tocopheryl radical derived from vitamin E can return to its reduced state as tocopherol by oxidizing vitamin C. Therefore, the interaction of vitamins E and C continuously restores the antioxidant function of vitamin E, maintaining the status of the latter [23].

The aim of this experiment was to investigate the effect of vitamin 25(OH)D_3_, E, or C, or a combination of all three, observed on growth performance, blood parameters, antioxidant status, and gene expression related to the inflammatory response and oxidative stress during a DON contamination in diets in weaned piglets. In addition, this study evaluated the effectiveness of the supplementation on these vitamins when a challenge with LPS is also implemented.

## 2. Results

### 2.1. Growth Performance

After 21 days of repetitive exposure to DON, piglets showed reduced body weight (BW; *p* < 0.001; Table 1), average daily gain (ADG; *p* < 0.001), and average daily feed intake (ADFI; *p* < 0.001). The vitamin 25(OH)D_3_, E, or C supplementation did not modify growth performance. Feed conversion ratio (FCR) was not impacted by DON contamination, regardless of the vitamin supplementation. Viscera weight relative to body weight did not differ between treatments while the value of the liver increased in piglets receiving DON_VitE-C (*p =* 0.088) and DON_Vit+ (*p* < 0.05) compared to DON.

### 2.2. LPS-Unchallenged Condition

#### 2.2.1. Blood Parameters

Under unchallenged conditions, DON (*p* < 0.001; Table 2) and DOM-1 (*p* < 0.001) concentrations in serum increased in piglets receiving DON. Interestingly, DON_Vit25-OHD decreased the serum levels of DOM-1 compared to DON alone (*p* < 0.05), but it did not modify any other blood parameters. Serum 25(OH)D_3_ concentrations were reduced for DON in comparison with CON (−62%; *p* < 0.05), and the addition of vitamins separately did not change this response. However, DON_Vit+ piglets had an increase in 25(OH)D_3_ levels compared to DON (+215%, *p* < 0.01). Plasma tocopherol concentration was enhanced in DON_VitE-C and DON_Vit+ piglets in comparison with DON (+86%; *p* < 0.05 and +134%, *p* < 0.001). Levels of vitamin C and 1,25(OH)_2_D_3_ were not impacted by any of the vitamin supplementation under the LPS-unchallenged condition. The DON contamination alone did not modify tocopherol.

The activity of GPx in plasma tended to be increased in DON piglets compared to CON (+49%; *p =* 0.064; Table 2), while total GSH in plasma had a tendency to be reduced in DON piglets compared to CON (−10%; *p =* 0.096). The other oxidation-related parameters were not changed with the diet contaminated with DON alone. Piglets consuming DON_Vit25-OHD (−37%; *p* < 0.05) and DON_VitE-C (−51%; *p* < 0.01) diets separately showed a decreased GPx activity in comparison with DON. The plasma MDA concentration tended to be decreased by DON_VitE-C compared to DON (−18%; *p =* 0.104). The activity of CAT has increased among DON_Vit+ piglets in comparison with DON (+62%, *p* < 0.05). The total GSH concentration had also a tendency to increase in piglets fed DON_Vit+ (+12%, *p =* 0.096). However, serum levels of TNF-α increased for DON_Vit+ compared to DON (+149%, *p* < 0.01).

#### 2.2.2. Intestinal Tissues

Under unchallenged conditions, piglets receiving DON_Vit+ had a higher MDA concentration in jejunal mucosa compared to either DON (+44%, *p* < 0.05; Table 3). The VDR gene expression was reduced for DON_Vit+ piglets in comparison with DON (−66%, *p* < 0.01). DON contamination alone and single-vitamin supplementation did not modify the other antioxidant concentrations (CAT, SOD, or GPx) in the intestine and the expression of genes (TNF-α and Nrf2) under the unchallenged LPS condition.

#### 2.2.3. Liver Tissues

Piglets receiving DON_VitE-C tended to have a decrease in MDA concentration in the liver compared to DON (−25%; *p* = 0.082; Table 4), and so did those receiving DON_Vit+, in comparison with DON (−27%, *p =* 0.063). The DON contamination alone did not modify the expression of any of the genes (IL-10, TLR4, VDR, TNF-α, Nrf2, or CYP2R1) or any other antioxidants (CAT, GPx, or SOD). Piglets receiving DON_Vit+ tended to have an increase in TNF-α gene expression compared to DON (+52%, *p =* 0.073). The single-vitamin supplementation did not change the other liver parameters (CAT, GPx, or SOD) or expression of genes (TLR4, VDR, TNF-α, Nrf2, or CYP2R1) in the unchallenged group.

#### 2.2.4. Mesenteric Lymph Nodes and Kidney Gene Expression

Under the LPS unchallenged condition, the TLR4 gene expression in mesenteric lymph nodes tended to be increased by DON compared to CON (+116%; *p =* 0.060; Table 5). There were no other impacts of DON contamination, and the vitamin intake did not affect the expression of genes in the mesenteric lymph nodes (IL-10, TLR4, VDR, TNF-α, or Nrf2). In the kidney, the CYP27B1 gene expression had a tendency to decrease for DON_Vit25-OHD piglets in comparison with DON (+55%; *p =* 0.091; Table 5). There was no impact of DON contamination and no other effects of the vitamin supplementation on the expression of genes in the kidney (CYP27B1, VDR).

### 2.3. LPS-Challenged Piglets

#### 2.3.1. Blood Parameters

After LPS challenge, piglets receiving DON had enhanced DON (*p* < 0.001; Table 6) and DOM-1 (*p* < 0.001) concentrations in the serum compared to CON. Serum levels of DON also tended to be higher for animals fed DON_VitE-C in comparison with DON (*p =* 0.059). Serum 25(OH)D_3_ concentration was reduced by DON contamination compared to CON (−72%; *p* < 0.01), but the addition of 25(OH)D_3_ increased it in comparison with DON in DON_Vit25-OHD piglets (255%; *p* < 0.05). Tocopherol level was increased with VitE-C supplementation (118%; *p* < 0.001) and DON_Vit+ (+128%, *p* < 0.001) compared to DON. Concentrations of 1,25(OH)_2_D_3_, vitamin C, calcium, and phosphorus were not impacted by any of the vitamin intake after the challenge. Tocopherol, vitamin C, and 1,25(OH)_2_D_3_ levels were not changed with a diet contaminated with DON alone after a challenge with LPS.

TNF-α concentration was upregulated in DON piglets compared to CON (+21%; *p* < 0.05; Table 6). Animals fed DON_Vit25-OHD tended to have an increase in GPx activity in comparison with DON (+39%; *p =* 0.056). DON contamination alone and different vitamin supplements did not modify the other blood parameters that are related to oxidation after LPS stimulation (CAT, SOD, total GSH, MDA, or IL-10).

#### 2.3.2. Intestinal Tissues

After the piglets were challenged with LPS, SOD activity in the jejunum was decreased by DON compared to CON (−42%; *p* < 0.05; Table 7). DON contamination alone did not modify any expression of genes. After LPS challenge, TNF-α gene expression was reduced for DON_VitE-C (−47%; *p* < 0.05) and for DON_Vit+ piglets in comparison with DON (−68%; *p* < 0.01). There was also a reduction in IL-10 gene expression among animals fed DON_Vit+ compared to DON (−68%; *p* < 0.05). Gene expression of Nrf2 tended to decrease in DON_VitE-C piglets in comparison with DON (−48%; *p =* 0.095). Vitamin supplements did not change the other intestinal parameters (CAT, GPx, or MDA) or gene expressions (IL-10, TLR4, or VDR) after LPS stimulation.

#### 2.3.3. Liver Tissues

After LPS challenge, MDA concentration in liver was increased for DON_Vit25-OHD piglets compared to DON (+64%; *p* < 0.001; Table 8). CAT activity tended to be decreased for DON_VitE-C piglets in comparison with DON (−31%; *p =* 0.096). Levels of other antioxidants and oxidants (CAT, GPx, SOD, and MDA) were not changed with a diet contaminated with DON alone. Nrf2 gene expression in the liver had a tendency to be reduced by DON contamination compared to CON (−63%; *p =* 0.066). Gene expression of IL-10 (+341%; *p* < 0.001) and VDR (+305%; *p* < 0.05) has increased with VitE-C supplementation in comparison to DON. There was also an increase in VDR (+274%; *p* < 0.05) and CYP2R1 (+55%; *p =* 0.086) gene expression in animals receiving DON_Vit+. DON contamination alone did not modify the expression of other genes (IL-10, TLR4, VDR, TNF-α, or CYP2R1), and the 25(OH)D_3_ supplementation did not impact any liver gene expression (IL-10, TLR4, VDR, TNF-α, Nrf2, or CYP2R1).

#### 2.3.4. Mesenteric Lymph Nodes and Kidney Gene Expression

After LPS challenge, piglets receiving DON-contaminated feed tended to have an increased IL-10 gene expression in the mesenteric lymph nodes compared to CON (+56%; *p =* 0.063; Table 9). Gene expression of IL-10 was then reduced by DON_VitE-C (−41%; *p* < 0.05) and had also a tendency to be reduced with DON_Vit25-OHD (−36%, *p =* 0.069) and DON_Vit+ (−33%, *p =* 0.100) in comparison with DON. There was a reduction in TNF-α gene expression in these nodes for piglets fed DON_Vit25-OHD (−43%; *p* < 0.05), DON_VitE-C (−55%; *p* < 0.01), and DON_Vit+ (−44%; *p* < 0.05) compared to DON. Gene expression of TLR4 in the lymph nodes also tended to be decreased by DON_VitE-C compared to DON (−44%; *p =* 0.076). In the kidneys, there was a decrease in both CYP27B1 (−55%; *p* < 0.05) and VDR (−41%; *p* < 0.05) gene expression with DON contamination compared to CON. Expression of CYP27B1 also tended to increase with DON_VitE-C in comparison with DON (+95%; *p =* 0.061). After LPS stimulation, there were no other effects of DON contamination and vitamin supplementation on the mesenteric lymph nodes and kidney gene expression.

## 3. Discussion

In this study, DON was the only mycotoxin present in significant quantity (DON, 12.48 mg/kg from naturally contaminated wheat; aflatoxins < 1.0 ppb; zearalenone < 0.03 ppm; fumonisin < 0.1 ppm; ochratoxins < 0.003 ppm; T-2 < 0.06). This mycotoxin is constantly present in pigs feed and is very challenging to eliminate, as it withstands high temperatures and low pH levels [2]. Therefore, the objectives of this study were to investigate the potential of vitamin 25(OH)D_3_, E, and C supplements, either individually or in combination, to alleviate the effects of DON contamination of the diet on growth performance, inflammatory response, and antioxidant status of piglets. The current design makes it possible to evaluate the effect of vitamin supplements, but only in the presence of DON contamination. It is worth mentioning that the concentrations of DON in the experimental diets is approximately fivefold higher than the recommended level of 1.0 mg/kg in Canada corresponding to an acute response. Also, as the environment of livestock can be contaminated with various bacteria and intestine lumen is a natural reservoir of commensal Gram-negative bacteria, piglets are likely to be exposed to LPS. The latter can interfere with the immune system and cause inflammatory reaction [8]. Previous works have shown that acute LPS challenge with DON in feed had synergistic effects on the response of the innate immune system and systemic circulation [11,24]. However, this endpoint is not always observed in DON-contaminated diets for pigs with intravenous administration of LPS on inflammation parameters [8,25]. Another objective of the current study was to evaluate the effect of these supplements on the inflammatory response and the antioxidant status during acute inflammation induced by LPS.

### 3.1. Impact of DON Contamination and Vitamin 25(OH)D_3_, E, and C Supplements on Growth Performance

The DON-contaminated diet reduced the ADFI and ADG by 39% and 36%, respectively, without modifying feed efficiency compared to CON after 21 days. The weight of piglets receiving DON was decreased after this period, but not the weight of the liver and intestinal viscera relative to body weight. This anorexic effect of DON has been described in previous studies [4,26,27], and led to reduced ADG due to a decrease in energy and nutrient intakes. One of the mechanisms that induce anorexia is linked to the neurotoxic effect of DON on serotonin secretion into the plasma, which was found to be increased by DON (0.25 mg/kg BW) in the plasma of minks [28] and in specific regions of the brain among piglets receiving DON-contaminated feed (2.2 mg/kg BW) [29]. However, there was no effect of a vitamin 25(OH)D_3_, E, or C supplementation on growth performance. Vitamin D is known to improve piglets’ growth and maintenance of functional skeleton, especially at a young age [30,31], while vitamin E has not been efficient to improve growth performance in other studies [21,32]. In this study, these vitamins alone and their combination did not have an impact on the mechanism associated with control of feed intake (serotonin secretion) in piglets receiving DON-contaminated diet. On the contrary, a combination of vitamins (D_3_, E, and C) has proven to be effective to improve growth performance when they were supplemented in a mixture of preservation components, including citric acid, potassium sorbate, sodium metabisulfite, vitamin A acetate, and amino acids, in piglets that were fed 4.0 mg/kg of DON for 14 days [5].

### 3.2. Blood Vitamins in Response to DON and Vitamin Supplementation

DON contamination decreased serum 25(OH)D_3_ concentrations after 21 days compared to CON in piglets both exposed to LPS or not. This was also reported in our previous study, where piglets receiving either vitamin D_3_ (2200 IU per kg) or 25(OH)D_3_ (2000 IU in the form of 25(OH)D_3_/per kg) supplementation still had a decrease in serum levels of 25(OH)D_3_ and calcitriol with DON contamination (4.9 mg/kg) [27]. On the other hand, the combination of vitamin 25(OH)D_3_ with vitamin E-C supplementation along with a DON-contaminated diet without LPS increased serum 25(OH)D_3_ concentration in piglets in comparison with DON alone or individually providing these vitamins. This is maybe due to an interaction between vitamins; Sergeev et al. [33] showed that VitC plays a critical role in vitamin D metabolism by affecting vitamin D endocrine system at both level of 1,25(OH)_2_D_3_ formation in kidneys and its binding receptor in target tissues in guinea pigs. Also, Bergstrom et al. [34] have shown that serum 1,25(OH)_2_D_3_ and 24,25(OH)_2_D_3_ were numerically greater for pigs fed vitamin C. But neither DON nor vitamin supplements modified calcitriol level. In this study, after a LPS challenge, concentration of 25(OH)D_3_ in serum was still decreased in piglets receiving DON-contaminated diets. However, the supplementation of 25(OH)D_3_ led to a significant increase in serum 25(OH)D_3_ level, suggesting that this intake during immune challenge can enhance vitamin D status to fulfill its immunomodulatory role against pathogenic molecules like LPS [35].

Vitamin E plays a role in protecting the components of cellular membranes including intestinal membrane [13]. Tocopherol concentration was increased by vitamin E-C supplementation in piglets both challenged with LPS or not; this can be attributed to the vitamin C’s potential to recycle tocopheryl radical into antioxidant form [23]. This increase in α tocopherol level after 21 days of vitamin E and C supplementation can indicate that the vitamin E was properly absorbed by piglets even with the lower feed intake with DON [15]. Additionally, the levels of tocopherol in the blood of piglets challenged or not with LPS were elevated by the combination of all three vitamins, indicating that the antioxidant potency of vitamin C to recycle tocopherol radicals might be facilitated by vitamin 25(OH)D_3_ supplements when piglets are exposed to DON.

### 3.3. Oxidative Stress, Inflammatory, and Vitamin D Response to DON

The DON contamination alone without LPS reduced the total concentration of GSH, an oxygen-derived free radical scavenger [36], and increased the antioxidant GPx activity in the blood. GPx is responsible for regulating the oxidant status by removing hydroperoxides and by oxidizing GSH [37]. Therefore, DON appears to have induced systemic oxidative stress in piglets after 21 days of repetitive exposure, as it was previously documented in pigs [7,38] and pig intestinal explants [39]. However, MDA level, a product of lipid peroxidation [40] in the blood and tissues (liver and jejunal mucosa), was not modified by DON contamination. Previous studies found mixed results; for instance, plasma MDA concentration was unaffected by DON-contaminated diet (4 mg/kg) in pigs after 14 days [21], while higher plasma H_2_O_2_ and MDA levels were reported in piglets fed this same diet (4 mg/kg) after 30 days [38]. The difference in MDA concentrations can be due to the time of exposure of 21 days and the different doses of DON used. Co-contamination with other mycotoxins can also induce lipid peroxidation, which was not the case in this study [41], as the other mycotoxins evaluated were below the level of detection. These findings were not observed under LPS-challenged conditions, but the antioxidant activity of SOD in the intestine and Nrf2 gene expression in the liver were decreased as a result of DON contamination. The antioxidant enzyme SOD can remove superoxide free radicals [42], while Nrf2 modulates target genes encoding detoxifying enzymes, like SOD, and antioxidant proteins in response to oxidative stress [43].

In the mesenteric lymph nodes, TLR4 gene expression, which plays a role in inflammatory signaling to protect against epithelial injury and bacterial invasion [44], tended to be increased by DON without the LPS stimulation, suggesting that DON exposure triggered inflammation. Piglets exposed to DON under an LPS challenge also had an increased IL-10 expression in mesenteric lymph nodes and an elevated serum TNF-α concentration. This implies that DON induced a markedly increased inflammatory response in piglets exposed to a challenge with LPS. In the kidneys, both CYP27B1 and VDR gene expression were decreased by DON contamination in piglets under LPS stimulation. This modification of expression for CYP27B1 was not associated with changes in calcitriol concentration, but it suggests that DON may alter the response associated with LPS challenge by reducing the conversion of 25(OH)D_3_ into 1,25(OH)_2_D_3_. As for VDR, it is important in the inflammatory response, since it binds to calcitriol to mediate its biological effects in immune cells [45]. The effect of DON on the vitamin D metabolism has not been the subject of a lot of studies, and these effects are probably indirectly associated with inflammation and immunomodulatory effect of this vitamin [46].

### 3.4. Effect of Vitamin E, C, and 25(OH)D_3_ Supplementation during DON Contamination on Oxidative Stress and Inflammatory Response

The addition of either vitamin E, C, or 25(OH)D_3_ in unchallenged piglets decreased GPx activity increased by DON. MDA concentration also tended to decrease in the blood and liver tissues when vitamins E and C were added, outlining the capacity of these two vitamins to reduce oxidative stress. Hepatic CAT activity and intestinal Nrf2 gene expression were decreased as well by the addition of both vitamins under LPS stimulation. CAT catalyzes the decomposition of hydrogen peroxide (H₂O₂), a ROS, into water and oxygen [47]. Thus, this reduction in CAT in LPS challenged piglets receiving vitamin E and C may be the result of reduced H₂O₂ production. However, H₂O₂ concentration was not evaluated in this study to confirm this theory. This study showed that vitamin E-C supplementation has a potentially protective effect against DON- and LPS-induced oxidative stress. This was observed in male weaner pigs under the stress of experimental *E. coli* infection, in part due to vitamin E’s role to prevent cellular oxidative damage [48]. However, liver MDA concentration and GPx blood levels were increased by DON_Vit25-OHD under a challenge with LPS. This indicates oxidative stress conditions. Vitamin 25-OH-D_3_ is known to influence the immune system [46] and could interfere with the normal immune response during a challenge with DON and LPS. Indeed, DON_Vit25-OHD treatment under a challenge with LPS increased the 25-OH-D_3_ status and decreased TNF-α and IL-10 genes expression in lymph nodes. Furthermore, the combination of vitamin 25(OH)D_3_ with vitamins E-C was more effective in reducing the hepatic oxidation by MDA, and in upregulating the antioxidant activity of CAT and total GSH in plasma in unchallenged piglets. Thus, combining vitamin 25(OH)D_3_ with vitamins E and C may confer protection against oxidative damage in hepatic tissues and improved the antioxidant response in piglets fed a diet contaminated with DON. Le Thanh et al. (2016) previously observed that only a combination of non-enzymatic antioxidants (vitamins A, E, and C; quercetin; organic selenium; and GSH) lowered DON- and ZEN-induced oxidation and systemic oxidative stress among piglets. Indeed, individually providing these antioxidants in the diet did not reduce the oxidative stress caused by DON and ZEN contamination. They also showed that a combination of vitamins A, E, and C reduced the hepatic MDA concentration in piglets [7]. On the other hand, the level of MDA in jejunum was increased with DON_Vit+. Again, this could be the result of an increased 25-OH-D_3_ blood status, which could influence the immune cell function and cytokine production [46], potentially leading to enhanced ROS production associated with the inflammatory process [2]. Oxidative stress in the jejunal mucosae can lead to alteration of the epithelial barrier, and thus modification nutrient absorptions, especially ions like calcium and phosphorus [49,50].

Indeed, blood CAT activity and pro-inflammatory cytokine TNF-α level were also increased by DON_Vit+. TNF-α hepatic gene regulation was also increased by the combination of vitamins 25(OH)D_3_, E, and C with DON-contaminated feed. On the contrary, in the intestinal mucosa under an LPS challenge, expression of TNF-α and IL-10 genes were reduced in piglets fed DON_Vit+, as opposed to their numerical elevation by DON. Expression of TNF-α in the jejunum was also reduced by DON_VitE-C. The combination of vitamins 25(OH)D_3_, E, and C could have intestinal anti-inflammatory effect during the immune stimulation with LPS, but not with only DON contamination. Indeed, IL-10 is an anti-inflammatory cytokine [24] and TNF-α plays an important role in regulating cell survival, proliferation, and death [51]. Additionally, vitamin E-C supplementation increased the expression of IL-10 in the liver among piglets under LPS stimulation. Both of these vitamins could be efficient for decreasing liver inflammation, which was reported in porcine hepatocyte cells when exposed to LPS (1 μg/mL) and higher DON exposure (500 nM and 2000 nM), where TNF- α and IL-10 mRNA expressions were upregulated [24]. The supplementation of vitamins E and C also reduced IL-10, TNF-α, and TLR4 gene expression while the combination of all three vitamins reduced IL-10 and TNF-α in mesenteric lymph nodes of piglets exposed to DON and LPS. During inflammatory reaction induced by LPS, these nodes can expand the number of neutrophils, produced in response to various cytokines and TLR ligands that are then recruited to sites of inflammation to elicit an immune response [52]. Adding vitamin E-C and 25(OH)D_3_ supplementation in combination prevented the increase in inflammatory markers with DON contamination and LPS stimulation.

Linked to the vitamin D metabolism, the VDR gene expression in the intestine was lowered in the DON_Vit+ group, possibly indicating reduced biological activity of calcitriol in the intestinal mucosa. On the contrary, among piglets under an LPS challenge, vitamin E-C supplementation and the combination of vitamins 25(OH)D_3_, E, and C increased the VDR gene expression in the liver compared to DON alone by at least 2.5-fold. VDR mediates the activity of many immune-related genes, including antimicrobial peptides cathelicidin and β-defensin, and biological action of calcitriol to stimulate the production of these molecules by epithelial cells [35]. Still in the liver, DON_Vit+ also had the tendency to increase gene expression of CYP2R1, responsible for hydroxylating vitamin D_3_ into 25(OH)D_3_ in LPS-challenged piglets. In the kidneys, the CYP27B1 gene expression tended to decrease in the DON_Vit25-OHD group compared to DON. Interestingly, vitamin E-C supplementation tended to increase the expression of CYP27B1 in the kidneys of piglets under a challenge with LPS. The vitamins thus have a potential anti-inflammatory role, probably linked to a decrease in ROS production [53], while increasing the regulation of genes in the kidneys associated with calcitriol during an inflammatory stimulation.

## 4. Conclusions

In summary, chronic exposure to DON contamination (5.1 mg/kg) over 21 days induced anorexia, leading to the reduced growth of piglets. Vitamin E, C, and 25(OH)D_3_ supplementation did not improve growth performance, indicating a limited effect on the mechanisms controlling the lower feed intake, including serotonin. DON contamination also induced oxidative stress and immune response in their circulatory and lymphatic systems along with a decrease in the vitamin D status. Supplementation with 25(OH)D_3_ alone in DON-contaminated feed failed to restore the vitamin D status. In contrast, vitamin E-C supplementation reduced both circulating and hepatic oxidative stress. In piglets under LPS challenge, vitamins E and C supplementation reduced intestinal, hepatic, and lymphatic markers of inflammation and protected against increased oxidative stress induced by both DON and LPS. However, the combination of vitamins 25(OH)D_3_, E, and C turned out to be the most promising as it demonstrated a potential in modulating antioxidant status and inflammatory responses, suggesting a protective role against oxidative damage and inflammation induced by DON contamination and LPS challenge in piglets.

## 5. Materials and Methods

### 5.1. Animals and Feeding Trials

The experiment was performed at the Centre de recherche en sciences animales de Deschambault (Quebec, Canada) and followed the guidelines of the Canadian Council on Animal Care (2009), and the protocol (2018-057) was approved by the Institutional Animal Care and Use Committee of Université Laval. All diets fulfilled the NRC requirements (Table 10) [54]. Fifty-four castrated male piglets ([Yorkshire x Landrace] × Duroc) weaned at 21 days of age (7.8 ± 0.14 kg) were distributed in 27 pens (two piglets/pen), depending on their weight at weaning after one week of acclimation with a commercial diet (Agri-Marché, St-Isidore, QC, Canada). Piglets then received one of the following 5 treatments: control treatments (CON) and DON-contaminated feed (DON, 5.1 mg/kg from naturally contaminated wheat; aflatoxins < 1.0 ppb; zearalenone < 0.03 ppm; fumonisin < 0.1 ppm; ochratoxin < 0.003 ppm; T-2 < 0.06). The DON treatments were supplemented with either 25-hydroxyvitamin D_3_ (DON_Vit25-OHD, 2000 IU in the form of 25(OH)D_3_/kg [0.05 mg/kg, Hy-D^®^, DSM)]), vitamins E and C (DON_VitE-C, 120 IU/kg in the form of α-tocopherol and 200 mg/kg in the form of ascorbic acid [Stay-C^®^50, DSM)]), or with all three vitamins (DON_Vit+, 25(OH)D_3_/kg with vitamin E and C) [55]. Piglets were distributed in those 5 treatments with either 5 or 6 repetitions (blocks) per treatment according to randomization method [55]. The CON and DON treatments had 6 repetitions while DON_Vit25-OH-D_3_, DON_VitE-C, and DON_Vit+ were repeated 5 times. Piglets received the experimental diets for 21 days and were fed ad libitum throughout the trial. Piglets were weighed at the beginning and the end of the trial and feed intake was evaluated after 7 days and 21 days per pen. At day 21, one piglet from each pen received intraperitoneal LPS injection (20 µg/kg BW, Sigma-Aldrich Canada, Oakville, ON, Canada) to induce acute inflammatory reaction 3 h prior to euthanasia. Blood samples were taken from each piglet just before euthanasia that was performed using a non-penetrating captive bolt stunner. Liver, intestinal jejunal mucosa, mesenteric lymph nodes, and left kidney tissue were collected, snap-frozen in liquid nitrogen, and kept at −80 °C until assessment.

### 5.2. Laboratory Analysis

#### 5.2.1. Blood and Tissue Analysis

Blood samples were taken from all piglets by jugular venipuncture (serum and heparin plasma tubes; BD Canada, Mississauga, ON, Canada). Plasma and serum tubes were centrifuged at 2000× *g* at 4 °C for 15 min. Supernatants were collected and kept frozen at −20 °C until analysis. Serum levels of DON and DOM-1 were measured by HPLC method [5]. Concentrations of 25(OH)D_3_ (Signalway Antibody, College Park, MD, USA) were determined in plasma by the sandwich ELISA method according to manufacturer instructions. Each well was treated with 40 μL of sample diluent and 10 μL of the sample. After incubation for 30 min at 37 °C, the plate was washed and HRP conjugate reagent (50 μL) was added to each well, except the blanks, and the plate was incubated again. After another wash, 50 μL of Chromogen Solution A and 50 μL of Chromogen Solution B were added to each well, and the plate was incubated for 10 min at 37 °C in the dark. The reaction was stopped by adding 50 μL of Stop Solution to each well, and absorbance was measured at 450 nm within 15 min. Concentrations of 1,25(OH)_2_D_3_ (BioVendor, Brno, Czech Republic) were determined in serum as previously described [48].

Total glutathione (GSH) plasma concentrations were assessed using the Glutathione Fluorescent Detection Kit (Arbor Assays, Ann Arbor, USA), as described by Le Thanh et al. [7]. The activity of GPx (Glutathione Peroxidase Assay Kit; Cayman Chemical Company, Ann Arbor, USA), CAT (Catalase Assay Kit; Cayman Chemical Company), and SOD (Superoxide Dismutase Assay Kit; Cayman Chemical Company) was measured in plasma samples. The GPx, CAT, and SOD activity were also evaluated in the intestine and liver following the manufacturer recommendations, where 50 mg of frozen intestinal mucosa or liver were homogenized in cold buffer. Quantification of cytokines was performed in serum with an enzyme-linked immunosorbent assay (ELISA) in order to have an overview of the systemic immune response to the treatments. TNF-α was quantified using the specific RayBio^®^ Porcine ELISA Kit (ELP-TNFalpha-001; RayBiotech, Peachtree Corners, USA). The samples (100 μL) were bound to the wells by the immobilized antibody on the microplate incubated for 2.5 h at room temperature with gentle shaking. Then, after washing, the biotinylated anti-porcine TNF-alpha antibody (100 μL) was added, and the plate was incubated again for 1 h. Finally, HRP-conjugated streptavidin (100 μL) and TMB substrate solution (100 μL) were added to the wells after properly washing the wells, followed by an incubation of 45 min and 30 min, respectively. Finally, 50 μL of Stop Solution was added to each well, and the absorbance was read immediately at 450 nm. As for IL-10, the ELISAs were conducted with the Quantikine^®^ ELISA kit (P1000) (R&D Systems, Minneapolis, USA). Each well received 100 μL of Assay Diluent RD1W and 100 μL of standard, control, or sample, then the microplate was incubated for 2 h at room temperature on a shaker. After the washing steps, Porcine IL-10 Conjugate (200 μL) was added to the wells and incubated for another 2 h on the shaker. The substrate solution (120 μL) was added to the wells following a wash step, and incubated for 30 min at room temperature, protected from light. The reaction was stopped (120 μL of Stop Solution), and optical density was measured at 450 nm within 30 min.

Lipid peroxidation in the plasma, intestine, and liver was assessed via a 2-thiobarbituric acid (TBA) color reaction for malondialdehyde (MDA) according to the modified method of Ermis et al. (2005) [56]. Briefly, approximately 150 mg of intestinal or liver mucosa was homogenized in 1.5 mL of cold phosphate-buffered saline (pH 7.4) and centrifuged at 2000 × *g* for 5 min at 4 °C. Then, 200 μL of plasma or liver or jejunum lysate was mixed with 25 μL of butylated hydroxytoluene and 500 μL of trichloroacetic acid. After being left on ice for 1 h, the samples were centrifuged at 2000× *g* for 15 min at 4 °C and approximately 500 μL of the supernatant was taken. Quantities of 125 μL of 1% TBA and 37.5 μL of EDTA were added to the samples. The samples were then boiled at 100 °C in water for 15 min and cooled to room temperature for a few minutes. They were then put on ice for 5 min and centrifuged at 2000× *g* for 30 s at 4 °C.

#### 5.2.2. Gene Expression Analysis

Tissues sensitive to DON-induced oxidative damages, such as the liver, kidney, mesenteric lymphoid organs, and intestine [57], were selected to evaluate gene expression related to inflammation, oxidative stress, and vitamin D metabolism. The samples (50 mg) were homogenized in 1 mL of TRIzol^TM^ (Thermo Fisher Scientific, Carlsbad, CA, USA). Then, 200 μL of chloroform were added and these were centrifuged for 15 min at 12,000× *g* at 4 °C. The aqueous phase was transferred in a new tube, with 500 μL of isopropanol. After centrifugation for 10 min 12,000× *g* at 4 °C, isopropanol was removed, and it was replaced by 75% ethanol for a 5 min centrifugation at 7500× *g* at 4 °C. Fifty μL of DNase-free water was added to dilute the pellet. The concentration and integrity of extracted RNAs were assessed with the NanoDrop 1000 Spectrophotometer (Thermo Fisher Scientific, Wilmington, DE, USA) and by nucleic acid electrophoresis with the Agilent 2100 Bioanalyzer instrument (Agilent, Santa Clara, CA, USA). Reverse transcription was performed with the qScript Flex cDNA Synthesis Kit (Qiagen Beverly, Inc., Cummings, MA, USA) with a 1 μg/μL mRNA concentration. The relative mRNA abundance of genes known to be involved in oxidative stress and inflammation was quantified using real-time qPCR analysis. Table A1 provides the complete list of selected genes. The qPCR was carried out with 10 μL of PerfeCTa^®^ SYBR^®^ Green FastMix^®^ (Quanta Biosciences, Inc., Gaithersburg, MD, USA), 1 μL of cDNAs, 1 μL of designed primers, and 8 μL of RNAse-free water on the Lightcycler 480 (Roche, Basel, Switzerland). The PCR cycling conditions were 10 min at 95 °C, followed by 50 cycles for 10 s at 57 °C or 58 °C (for primer annealing), depending on the gene, and 20 s at 72 °C (for primer extension), and a melt curve step was set up at 72 °C for 10 s at 94 °C for 5 measures. A relative standard curve was established by serial dilutions of a cDNA pool to determine the mRNA expression levels.

VDR gene expression was evaluated in all tissues. The hepatic gene expression of CYP2R1 (25-hydroxylase, catalyzes the conversion of vitamin D_3_ into 25(OH)D_3_) was assessed as well. In the liver, mesenteric lymph nodes, and jejunum mucosa, IL-10 (interleukin 10, an anti-inflammatory cytokine), TNF-α (tumor necrosis factor-alpha, a cytokine regulating the inflammatory response), TLR4 (Toll-like receptor 4, has a role in the activation of the innate immune system), and Nrf2 (nuclear factor erythroid 2-related factor 2, a regulator of cellular resistance to oxidants) gene expressions were also estimated. The CYP27B1 (1α-hydroxylase, hydroxylation of 25(OH)D_3_ into 1-25(OH)_2_D_3_) gene expression was assessed in the kidney. Values were then normalized with three housekeeping genes, GAPDH, β-actin, and HPRT.

### 5.3. Statistical Analyses

All the results were evaluated using the Glimmix procedure on SAS (SAS studio 2021; SAS Inst., Inc. Cary, NC, USA) with the five different treatments as the fixed effect with the following contrasts to compare: CON versus DON (DON effect), DON versus DON_Vit25-OHD (vitamin D effect), DON versus DON_VitE-C (vitamin E and C effect), and DON versus DON_Vit+ (vitamin + effect). Growth performance including average daily feed intake (ADFI), average daily gain (ADG), and feed conversion ratio (FCR) were analyzed with a pen of 2 piglets as the experimental unit. Blood samples, tissue samples, and gene expression were analyzed for each piglet, receiving LPS stimulation or not, with the piglet as the experimental unit. Thus, the unchallenged LPS group was analyzed separately from the LPS-challenged group. A *p*-value less than 0.05 indicates a significant difference, whereas a *p*-value between 0.05 and 0.10 indicates a statistical trend.

## Figures and Tables

**Table 1:** Impact of DON contamination and vitamin supplementation on growth performances after a 21-day period ^1^.

Items		ADG, g/d	ADFI, g/d	FCR	Initial BW, kg	Final BW, kg	Viscera/BW, %	Liver/BW, %
Treatments	*n* =							
CON	12	553	745	1.35	7.82	19.7	11.7	3.14
DON	12	336	477	1.41	8.10	15.4	12.4	3.16
DON_Vit25-OHD	10	364	476	1.32	7.66	15.6	11.6	3.06
DON_VitE-C	10	342	468	1.30	7.48	14.9	12.2	3.35
DON_Vit+	10	376	516	1.40	7.81	15.8	12.0	3.42
SEM		32.7	37.9	0.05	0.32	0.81	0.54	0.08
Contrasts (*p* value ^2^)								
DON effect		<0.001	<0.001	0.321	0.490	<0.001	0.303	0.909
Vitamin D effect		0.455	0.875	0.139	0.316	0.816	0.285	0.406
Vitamin E-C effect		0.816	0.984	0.111	0.156	0.628	0.791	0.088
Combined Vit+ effect		0.429	0.524	0.887	0.502	0.732	0.589	<0.05

CON: control diet; DON: deoxynivalenol; DON_Vit25-OHD: deoxynivalenol with 25-hydroxyvitamin D_3_; DON_VitE-C: deoxynivalenol with vitamins E and C; DON_Vit+: deoxynivalenol with 25-hydroxyvitamin D_3_ and vitamins E and C; BW: body weight; ADFI: average daily feed intake; ADG: average daily gain; FCR: feed conversion ratio. ^1^ Values are expressed as least squares means. ^2^ *p*-value less than 0.05 indicates a significant difference; *p*-value between 0.05 and 0.10 indicates a statistical trend.

**Table 2:** Effects of DON contamination and vitamin supplementation on blood parameters after a 21-day period ^1^.

Treatments	CON	DON	DON_Vit-25OHD	DON_VitE-C	DON_Vit+	SEM	*p*-Value ^2^			
							Effects			
Items	*n* = 6	*n* = 6	*n* = 5	*n* = 5	*n* = 5		DON	Vitamin D	Vitamins E and C	Combined Vit+
DON, ng/mL	0.21	23.55	23.02	26.00	27.89	3.15	<0.001	0.902	0.571	0.321
DOM-1, ng/mL	0.00	4.68	3.32	4.54	4.05	0.46	<0.001	<0.05	0.825	0.320
25(OH)D_3_, ng/mL	11.33	4.28	6.46	6.15	13.48	2.43	<0.05	0.543	0.577	<0.01
1,25(OH)_2_D_3_, pg/mL	147	136	139	117	136	8.62	0.389	0.841	0.112	0.996
Tocopherol, mg/L	4.83	3.63	3.92	6.76	8.48	0.85	0.290	0.808	<0.05	<0.001
Vitamin C	60.8	75.5	59.4	88.7	60.4	12.8	0.384	0.365	0.453	0.395
CAT, μM/min/mL	91.4	94.1	82.2	127	152	17.5	0.911	0.620	0.182	<0.05
SOD, U/mL	2.83	3.56	2.80	3.43	4.33	0.54	0.301	0.304	0.855	0.298
GPx, nM/min/mL	204	303	191	149	256	39.4	0.064	<0.05	<0.01	0.386
Total GSH, μM	7.61	6.83	7.04	6.97	7.65	0.35	0.096	0.662	0.761	0.096
MDA, μM	5.68	6.54	5.39	5.38	5.62	0.51	0.203	0.107	0.104	0.196
TNF-α, pg/mL	17.1	17.7	12.9	12.1	44.1	8.8	0.961	0.694	0.644	<0.05
IL-10, pg/mL	7.39	8.27	8.50	7.35	6.83	2.43	0.783	0.944	0.783	0.666

CON: control diet; DON: deoxynivalenol; DON_Vit25-OHD: deoxynivalenol with 25-hydroxyvitamin D_3_; DON_VitE-C: deoxynivalenol with vitamins E and C; DON_Vit+: deoxynivalenol with 25-hydroxyvitamin D_3_ and vitamins E and C; DOM-1: deepoxy deoxynivalenol; 25(OH)D_3_: 25-hydroxyvitamin D_3_; 1,25(OH)_2_D_3_: calcitriol; CAT: catalase; SOD: superoxide dismutase; GPx: glutathione peroxidase; GSH: glutathione; MDA: malondialdehyde; TNF-α: tumor necrosis factor-alpha; IL-10: interleukin 10. ^1^ Values are expressed as least squares means. ^2^ *p*-value less than 0.05 indicates a significant difference; *p*-value between 0.05 and 0.10 indicates a statistical trend.

**Table 3:** Effects of DON contamination and vitamin supplementation on oxidative markers and gene expression in jejunum after a 21-day period ^1^.

	CON	DON	DON_Vit25-OHD	DON_VitE-C	DON_Vit+	SEM	*p*-Value ^2^			
							Effects			
Items	*n* = 6	*n* = 6	*n* = 5	*n* = 5	*n* = 5	*n* = 6	DON	Vitamin D	Vitamins E and C	Combined Vit+
CAT, μM/min/mL/g	30.7	27.6	27.3	23.5	25.8	4.43	0.606	0.973	0.521	0.784
SOD, U/mL/g	24.8	33.4	29.4	20.6	34.9	5.64	0.252	0.604	0.109	0.842
GPx, nM/min/mL/g	5.38	7.66	6.96	7.47	5.47	1.12	0.130	0.668	0.901	0.163
MDA, μM/g	179	141	162	143	203	19.6	0.149	0.456	0.926	< 0.05
IL-10	0.73	0.95	0.82	0.60	1.13	0.17	0.356	0.597	0.161	0.475
TNF-α	0.71	1.32	1.32	0.90	0.93	0.33	0.183	0.993	0.376	0.417
TLR4	0.70	0.84	0.65	0.64	1.10	0.18	0.568	0.457	0.565	0.309
Nrf2	1.13	1.15	1.08	1.01	1.07	0.26	0.963	0.849	0.708	0.834
VDR	1.48	1.92	1.92	1.83	0.65	0.34	0.235	0.990	0.794	< 0.01

CON: control diet; DON: deoxynivalenol; DON_Vit25-OHD: deoxynivalenol with 25-hydroxyvitamin D_3_; DON_VitE-C: deoxynivalenol with vitamins E and C; DON_Vit+: deoxynivalenol with 25-hydroxyvitamin D_3_ and vitamins E and C; CAT: catalase; SOD: superoxide dismutase; GPx: glutathione peroxidase; GSH: glutathione; MDA: malondialdehyde; TNF-α: tumor necrosis factor-alpha; IL-10: interleukin 10; TLR4: Toll-like receptor 4; Nrf2: nuclear factor erythroid 2-related factor 2; VDR: vitamin D receptor.^1^ Values are expressed as least squares means. ^2^ *p*-value less than 0.05 indicates a significant difference; *p*-value between 0.05 and 0.10 indicates a statistical trend.

**Table 4:** Effect of DON contamination and vitamin supplementation on oxidation markers and gene expression in liver after a 21-day period ^1^.

	CON	DON	DON_Vit25-OHD	DON_VitE-C	DON_Vit+	SEM	*p*-Value ^2^			
							Effects			
Items	*n* = 6	*n* = 6	*n* = 5	*n* = 5	*n* = 5		DON	Vitamin D	Vitamins E and C	Combined Vit+
CAT, μM/min/mL/g	280	244	305	249	228	57.2	0.638	0.447	0.950	0.831
SOD, U/mL/g	7.84	5.52	9.39	5.35	9.57	1.82	0.334	0.131	0.947	0.115
GPx, nM/min/mL/g	6.82	8.60	7.83	7.42	7.45	1.67	0.417	0.738	0.606	0.617
MDA, μM/g	41.48	44.81	53.25	33.74	32.91	4.48	0.571	0.178	**0.082**	**0.063**
IL-10	0.95	0.91	0.78	0.66	1.15	0.20	0.882	0.643	0.374	0.399
TNF-α	1.07	0.90	1.06	1.04	1.37	0.18	0.487	0.537	0.584	**0.073**
TLR4	0.41	0.28	0.16	0.19	0.34	0.08	0.207	0.288	0.456	0.583
Nrf2	0.84	1.26	0.75	0.76	0.73	0.39	0.363	0.296	0.309	0.310
CYP2R1	1.29	1.17	1.22	1.26	1.12	0.22	0.664	0.865	0.760	0.885
VDR	0.43	0.54	0.30	0.30	0.51	0.11	0.482	0.136	0.139	0.833

CON: control diet; DON: deoxynivalenol; DON_Vit25-OHD: deoxynivalenol with 25-hydroxyvitamin D_3_; DON_VitE-C: deoxynivalenol with vitamins E and C; DON_Vit+: deoxynivalenol with 25-hydroxyvitamin D_3_ and vitamins E and C; CAT: catalase; SOD: superoxide dismutase; GPx: glutathione peroxidase; GSH: glutathione; MDA: malondialdehyde; TNF-α: tumor necrosis factor-alpha; IL-10: interleukin 10; TLR4: Toll-like receptor 4; Nrf2: nuclear factor erythroid 2-related factor 2; VDR: vitamin D receptor; CYP2R1: 25-hydroxylase. ^1^ Values are expressed as least squares means. ^2^ *p*-value less than 0.05 indicates a significant difference; *p*-value between 0.05 and 0.10 indicates a statistical trend.

**Table 5:** Effect of DON contamination and vitamin supplementation on gene expression in mesenteric lymph nodes and kidney after a 21-day period ^1^.

	CON	DON	DONVit_25-OHD	DON_VitE-C	DON_Vit+	SEM	*p*-Value ^2^			
							Effects			
Items	*n* = 6	*n* = 6	*n* = 5	*n* = 5	*n* = 5		DON	Vitamin D	Vitamins E and C	Combined Vit+
Lymph nodes										
IL-10	0.71	1.03	1.17	1.12	0.81	0.23	0.324	0.670	0.787	0.519
TNF-α	1.08	1.19	1.43	0.78	0.89	0.23	0.726	0.449	0.206	0.344
TLR4	0.69	1.49	1.34	1.26	0.90	0.31	0.060	0.740	0.598	0.178
Nrf2	1.02	1.18	0.84	1.07	0.92	0.22	0.586	0.299	0.719	0.392
VDR	0.99	1.08	1.17	1.00	0.72	0.22	0.762	0.738	0.791	0.234
Kidneys	***n* = 5**	***n* = 5**	***n* = 3**	***n* = 3**	***n* = 3**					
CYP27B1	0.78	0.76	0.34	0.53	0.41	0.19	0.937	0.091	0.330	0.149
VDR	0.84	0.72	0.91	0.71	0.82	0.14	0.471	0.320	0.941	0.611

CON: control diet; DON: deoxynivalenol; DON_Vit25-OHD: deoxynivalenol with 25-hydroxyvitamin D_3_; DON_VitE-C: deoxynivalenol with vitamins E and C; DON_Vit+: deoxynivalenol with 25-hydroxyvitamin D_3_ and vitamins E and C; CAT: catalase; SOD: superoxide dismutase; GPx: glutathione peroxidase; GSH: glutathione; MDA: malondialdehyde; TNF-α: tumor necrosis factor-alpha; IL-10: interleukin 10; TLR4: Toll-like receptor 4; Nrf2: nuclear factor erythroid 2-related factor 2; VDR: vitamin D receptor; CYP27B1: 1α-hydroxylase. ^1^ Values are expressed as least squares means. ^2^ *p*-value less than 0.05 indicates a significant difference; *p*-value between 0.05 and 0.10 indicates a statistical trend.

**Table 6:** Effect of DON contamination and vitamin supplementation after the lipopolysaccharide stimulation on blood parameters after a 21-day period ^1^.

	CON	DON	DON_Vit25-OHD	DON_VitE-C	DON_Vit+	SEM	*p*-Value ^2^			
							Effects			
Items	*n* = 6	*n* = 6	*n* = 5	*n* = 5	*n* = 5		DON	Vitamin D	Vitamins E and C	Combined Vit+
DON, ng/mL	1.50	18.31	19.01	27.44	20.46	3.38	**<0.001**	0.880	**0.059**	0.643
DOM-1, ng/mL	0.00	4.94	4.07	4.77	4.16	0.57	**<0.001**	0.270	0.831	0.321
25(OH)D_3_, ng/mL	12.97	3.65	12.95	4.56	7.92	2.49	**<0.01**	**<0.05**	0.790	0.220
1,25(OH)_2_D_3_, pg/mL	140	131	149	113	141	9.63	0.539	0.198	0.205	0.495
Tocopherol, mg/L	4.69	3.54	3.19	7.73	8.07	0.67	0.198	0.706	**<0.001**	**<0.001**
Vitamin C	86.5	93.4	66.7	79.8	138	25.2	0.834	0.442	0.694	0.208
CAT, μM/min/mL	103	112	81.9	126	76.0	15.8	0.672	0.176	0.522	0.109
SOD, U/mL	3.83	3.51	3.30	3.94	3.15	0.49	0.623	0.751	0.527	0.585
GPx, nM/min/mL	189	174	241	173	159	24.5	0.631	**0.056**	0.978	0.661
Total GSH, μM	7.48	7.44	7.20	6.87	6.79	0.37	0.935	0.634	0.262	0.203
MDA, μM	5.54	5.32	5.43	5.54	5.47	0.16	0.271	0.599	0.283	0.491
TNF, pg/mL	2640	3200	3040	3358	3096	174	**<0.05**	0.502	0.512	0.662
IL-10, pg/mL	33.9	73.2	52.3	101	80.3	23.8	0.215	0.524	0.399	0.828

CON: control diet; DON: deoxynivalenol; DON_Vit25-OHD: deoxynivalenol with 25-hydroxyvitamin D_3_; DON_VitE-C: deoxynivalenol with vitamins E and C; DON_Vit+: deoxynivalenol with 25-hydroxyvitamin D_3_ and vitamins E and C; DOM-1: deepoxy deoxynivalenol; 25(OH)D_3_: 25-hydroxyvitamin D_3_; 1,25(OH)_2_D_3_: calcitriol; CAT: catalase; SOD: superoxide dismutase; GPx: glutathione peroxidase; GSH: glutathione; MDA: malondialdehyde; TNF-α: tumor necrosis factor-alpha; IL-10: interleukin 10. ^1^ Values are expressed as least squares means. ^2^ *p*-value less than 0.05 indicates a significant difference; *p*-value between 0.05 and 0.10 indicates a statistical trend.

**Table 7:** Effect of DON contamination and vitamin supplementation after lipopolysaccharide stimulation on oxidation markers and gene expression in the jejunum after a 21-day period ^1^.

	CON	DON	DON_Vit25-OHD	DON_VitE-C	DON_Vit+	SEM	*p*-Value ^2^			
							Effects			
Items	*n* = 6	*n* = 6	*n* = 5	*n* = 5	*n* = 5		DON	Vitamin D	Vitamins E and C	Combined Vit+
CAT, μM/min/mL/g	25.3	28.9	27.4	21.9	25.9	3.85	0.476	0.777	0.191	0.568
SOD, U/mL/g	31.0	18.1	19.4	22.8	20.7	4.54	<0.05	0.852	0.479	0.690
GPx, nM/min/mL/g	5.69	6.76	6.96	6.06	8.09	1.12	0.466	0.898	0.648	0.388
MDA, μM/g	176	202	184	146	173	26.6	0.453	0.625	0.131	0.427
IL-10	1.48	2.19	1.59	1.44	0.70	0.43	0.213	0.311	0.214	<0.05
TNF-α	1.34	2.01	1.44	1.06	0.65	0.31	0.117	0.196	<0.05	<0.01
TLR4	1.68	1.78	1.79	1.43	1.01	0.33	0.826	0.983	0.447	0.105
Nrf2	1.05	1.68	1.28	0.88	0.94	0.34	0.160	0.392	0.095	0.122
VDR	0.66	0.97	0.88	1.10	0.74	0.17	0.176	0.714	0.562	0.331

CON: control diet; DON: deoxynivalenol; DON_Vit25-OHD: deoxynivalenol with 25-hydroxyvitamin D_3_; DON_VitE-C: deoxynivalenol with vitamins E and C; DON_Vit+: deoxynivalenol with 25-hydroxyvitamin D_3_ and vitamins E and C; CAT: catalase; SOD: superoxide dismutase; GPx: glutathione peroxidase; GSH: glutathione; MDA: malondialdehyde; TNF-α: tumor necrosis factor-alpha; IL-10: interleukin 10; TLR4: Toll-like receptor 4; Nrf2: nuclear factor erythroid 2-related factor 2; VDR: vitamin D receptor. ^1^ Values are expressed as least squares means. ^2^ *p*-value less than 0.05 indicates a significant difference; *p*-value between 0.05 and 0.10 indicates a statistical trend.

**Table 8:** Effect of DON contamination and vitamin supplementation after lipopolysaccharide stimulation on oxidation markers and gene expression in the liver after a 21-day period ^1^.

	CON	DON	DON_Vit25-OHD	DON_VitE-C	DON_Vit+	SEM	*p*-Value ^2^			
							Effects			
Items	*n* = 6	*n* = 6	*n* = 5	*n* = 5	*n* = 5		DON	Vitamin D	Vitamins E and C	Combined Vit+
CAT, μM/min/mL/g	315	333	286	231	243	43.0	0.747	0.434	0.096	0.138
SOD, U/mL/g	11.2	10.7	11.0	11.2	6.68	1.92	0.861	0.908	0.870	0.132
GPx, nM/min/mL/g	6.66	6.18	5.64	5.17	6.02	1.03	0.719	0.706	0.480	0.914
MDA, μM/g	37.7	32.4	53.1	26.8	25.4	3.63	0.277	<0.001	0.269	0.167
IL-10	1.52	0.78	1.01	3.44	1.63	0.45	0.214	0.704	<0.001	0.177
TNF-α	1.04	0.91	0.95	1.31	1.21	0.25	0.680	0.898	0.286	0.387
TLR4	7.16	5.30	4.74	9.61	6.93	1.98	0.473	0.835	0.122	0.550
Nrf2	2.38	0.88	1.18	1.78	1.56	0.57	0.066	0.697	0.257	0.390
CYP2R1	0.75	0.73	0.69	1.09	1.13	0.17	0.929	0.878	0.125	0.086
VDR	4.84	1.74	2.31	7.05	6.51	1.51	0.125	0.780	<0.05	<0.05

CON: control diet; DON: deoxynivalenol; DON_Vit25-OHD: deoxynivalenol with 25-hydroxyvitamin D_3_; DON_VitE-C: deoxynivalenol with vitamins E and C; DON_Vit+: deoxynivalenol with 25-hydroxyvitamin D_3_ and vitamins E and C; CAT: catalase; SOD: superoxide dismutase; GPx: glutathione peroxidase; GSH: glutathione; MDA: malondialdehyde; TNF-α: tumor necrosis factor-alpha; IL-10: interleukin 10; TLR4: Toll-like receptor 4; Nrf2: nuclear factor erythroid 2-related factor 2; VDR: vitamin D receptor; CYP2R1: 25-hydroxylase. ^1^ Values are expressed as least squares means. ^2^ *p*-value less than 0.05 indicates a significant difference; *p*-value between 0.05 and 0.10 indicates a statistical trend.

**Table 9:** Effect of DON contamination and vitamin supplementation after the lipopolysaccharide stimulation on gene expression in mesenteric lymph nodes and kidneys after a 21-day period ^1^.

	CON	DON	DON_Vit25-OHD	DON_VitE-C	DON_Vit+	SEM	*p*-Value ^2^			
							Effects			
Items	*n* = 6	*n* = 6	*n* = 5	*n* = 5	*n* = 5		DON	Vitamin D	Vitamins E and C	Combined Vit+
Lymph nodes										
IL-10	1.33	2.07	1.31	1.22	1.39	0.29	0.063	0.069	<0.05	0.100
TNF-α	1.49	2.08	1.19	0.93	1.16	0.28	0.130	<0.05	<0.01	<0.05
TLR4	1.16	1.83	1.29	1.03	1.25	0.32	0.119	0.221	0.076	0.191
Nrf2	1.33	1.28	1.63	1.01	1.06	0.23	0.862	0.273	0.394	0.487
VDR	0.90	1.33	1.20	1.38	1.94	0.35	0.321	0.778	0.925	0.212
Kidneys	*n* = 4	*n* = 5	*n* = 5	*n* = 5	*n* = 4					
CYP27B1	3.13	1.41	2.35	2.75	1.47	0.53	<0.05	0.177	0.061	0.928
VDR	1.95	1.15	1.63	1.27	1.56	0.28	<0.05	0.192	0.734	0.292

CON: control diet; DON: deoxynivalenol; DON_Vit25-OHD: deoxynivalenol with 25-hydroxyvitamin D_3_; DON_VitE-C: deoxynivalenol with vitamins E and C; DON_Vit+: deoxynivalenol with 25-hydroxyvitamin D_3_ and vitamins E and C; CAT: catalase; SOD: superoxide dismutase; GPx: glutathione peroxidase; GSH: glutathione; MDA: malondialdehyde; TNF-α: tumor necrosis factor-alpha; IL-10: interleukin 10; TLR4: Toll-like receptor 4; Nrf2: nuclear factor erythroid 2-related factor 2; VDR: vitamin D receptor; CYP27B1: 1α-hydroxylase. ^1^ Values are expressed as least squares means. ^2^ *p*-value less than 0.05 indicates a significant difference; *p*-value between 0.05 and 0.10 indicates a statistical trend.

**Table 10:** Composition of experimental diets.

	^a^ Control	DON	DON_Vit25-OHD	DON_VitE-C	DON_Vit+
Ingredient (%)					
Control, soft wheat	50.0				
Contaminated, soft wheat ^b^		50.0	50.0	50.0	50.0
Corn	14.1	14.1	14.1	14.1	14.1
Soybean meal	20.3	20.3	20.3	20.3	20.3
Hamlet soy protein (HP300)	3.03	3.03	3.03	3.03	3.03
Choice of white fat	2.05	2.05	2.05	2.05	2.05
Whey powder	7.00	7.00	7.00	7.00	7.00
Monocalcium phosphate	0.88	0.88	0.88	0.88	0.88
Limestone	1.16	1.16	1.16	1.16	1.16
Vitamin and mineral premix ^yz^	0.50	0.50	0.50	0.50	0.50
Salt	0.30	0.30	0.30	0.30	0.30
Lysine-HCl	0.45	0.45	0.45	0.45	0.45
DL-Methionine	0.13	0.13	0.13	0.13	0.13
L-Threonine	0.15	0.15	0.15	0.15	0.15
Calculated composition (%)					
Fat	8.40	8.40	8.40	8.40	8.40
Lactose	5.00	5.00	5.00	5.00	5.00
Crude protein	21.00	21.00	21.00	21.00	21.00
Calcium	0.85	0.85	0.85	0.85	0.85
Phosphorus digestible	0.42	0.42	0.42	0.42	0.42
SID lysine	1.31	1.31	1.31	1.31	1.31
Analyzed composition					
Protein, %	21.0	21.1	21.4	22.2	21.1
Calcium, %	0.94	1.05	1.27	1.14	0.85
Phosphorus, %	0.65	0.60	0.61	0.69	0.60
Vitamin A, IU/kg	2868	3303	3530	3453	2874
Vitamin E, IU/kg	35.0	33.0	27.0	112	111
Stay-C, mg/kg	---	---	---	282	288
Vitamin D, IU/kg	226	170	232	263	184
25(OH)D_3_	---	---	2864		2780
Deoxynivalenol, mg/kg	0.11	5.16	4.98	4.85	5.03

^a^ Control diet (0.11 mg/kg DON); DON-contaminated diet (5.16 mg/kg DON); DONVit25-OH-D_3_ diet (4.98 mg/kg DON); DON_VitE-C diet (4.85 mg/kg DON); DON_Vit+ diet (5.03 mg/kg DON). ^b^ Mycotoxin concentrations in contaminated wheat: deoxynivalenol 5.1 mg/kg from naturally contaminated wheat, aflatoxins < 1.0 ppb, zearalenone < 0.03 ppm, fumonisin < 0.1 ppm, ochratoxin < 0.003 ppm, T-2 < 0.06. ^z^ Provided per kilogram of diet: vitamin A palmitate 2000 IU; vitamin D3 200 IU; vitamin E acetate 16 IU; menadione sodium bisulfite 3.75 mg; thiamine HCl 1.0 mg; riboflavin 3.5 mg; niacin 30.0 mg; calcium pantothenate 15.0 mg; pyridoxine HCl 7.0 mg; biotin 0.5 mg; choline bitartrate 375 mg; vitamin B_12_ 25.0 µg. ^y^ Provided per kilogram of diet: Zn (as zinc carbonate) 100 mg; Fe (as ferric citrate) 100 mg; Cu (as cupric carbonate) 25 mg; I (as potassium iodate) 0.28 mg; Mn (as manganous carbonate) 46 mg; Se (as sodium selenite) 0.30 mg.

## Data Availability

The data sets generated in this study are available in this article and are available from the corresponding author upon request.

## Appendix A

**Table A1:** Primer sequences used for qPCR.

Gene	Primer Sequence (5′-3′)	Product Size, bp	GenBank Accession no.
Vitamin D			
CYP2R1	(F) TTCATCCCTTCTTGACTCCAAC	201	XM_003480731
	(R) TTTATCTGTCACCTGTCACCAC		
VDR	(F) AGGCTTCTTCAGACGGAGCAT	143	NM_001097414.1
	(R) ACTCCTTCATCATGCCGATGT		
CYP27B1	(F) TGGGCTCTCTATGAACTCTCTC	157	DQ295065.1
	(R) GTCTTAGCACTTCCTTGACCAC		
Inflammation			
IL-10	(F) ACTGAAGCATTCTAGGGAAACC	158	NM_214041.1
	(R) ATATCTCAGGGGAGAGGTACAG		
TNF-α	(F) GCCCACGTTGTAGCCAATGTCAAA	98	GC06P031543
	(R) TTGTCTTTCAGCTTCACGCCGTTG		
TLR4	(F) GACCTGAGCTTTAACTACCTG	160	NM_001113039.2
	(R) ATTTCCCGTCAGTATCAAGGTG		
*Oxidation*			
NrF2	(F) GGTGTTGGCAGTAGTCTAAAGG	215	XM_005671981.3
	(R) CACCTAGACCTTCGAACCATAG		
Reference			
GAPDH	(F) CCC CAA CGT GTC GGT TGT	91	XM_021091114.1
	(R) CTC GGA CGC CTG CTT CAC		
β-Actin	(F) CAT CAC CAT CGG CAA CGA	128	XM_003357928.4
	(R) GGA TGT CGA CGT CGC ACTT		
HPRT	(F) TTG TGG TAG GCT ATG CCC TTG ACT	117	NM_001032376
	(R) CTC AAC TTG AAC TCT CCT CTT AGG		
